# Stability of white matter changes related to Huntington’s disease in the presence of imaging noise: a DTI study

**DOI:** 10.1371/currents.RRN1232

**Published:** 2011-06-07

**Authors:** Hans-Peter Müller, Volkmar Glauche, Marianne J U Novak, Thao Nguyen-Thanh, Alexander Unrath, Nayana Lahiri, Joy Read, Miranda Julia Say, Sarah J Tabrizi, Jan Kassubek, Stefan Kloppel

**Affiliations:** ^*^Dept. of Neurology, University of Ulm, Ulm, Germany; ^†^Freiburg Brain Imaging, Department of Neurology, University Freiburg Medical Center, Germany; ^‡^Wellcome Trust Centre for Neuroimaging, UCL Institute of Neurology, London, UK; ^§^Department of Neuroradiology, University Medical Center Freiburg, Germany; ^#^Department of Neurodegenerative Disease, UCL Institute of Neurology, London, UK and ^¶¶^Freiburg Brain Imaging, Department of Psychiatry and Psychotherapy. University of Freiburg

## Abstract

Movement artifacts and other sources of noise are a matter of concern particularly in the neuroimaging research of movement disorders such as Huntington’s disease (HD). Using diffusion weighted imaging (DWI) and fractional anisotropy (FA) as a compound marker of white matter integrity, we investigated the effect of movement on HD specific changes in magnetic resonance imaging (MRI) data and how post hoc compensation for it affects the MRI results. To this end, we studied by 3T MRI: 18 early affected, 22 premanifest gene-positive subjects, 23 healthy controls (50 slices of 2.3 mm thickness per volume, 64 diffusion-weighted directions (b = 1000 s/mm2), 8 minimal diffusion-weighting (b = 100 s/mm2)); and by 1.5 T imaging: 29 premanifest HD, 30 controls (40 axial slices of 2.3 mm thickness per volume, 61 diffusion-weighted directions (b = 1000 s/mm2), minimal diffusion-weighting (b = 100 s/mm2)). An outlier based method was developed to identify movement and other sources of noise by comparing the index DWI direction against a weighted average computed from all other directions of the same subject. No significant differences were observed when separately comparing each group of patients with and without removal of DWI volumes that contained artifacts. In line with previous DWI-based studies, decreased FA in the corpus callosum and increased FA around the basal ganglia were observed when premanifest mutation carriers and early affected patients were compared with healthy controls. These findings demonstrate the robustness of the FA value in the presence of movement and thus encourage multi-center imaging studies in HD.

## **Introduction**

White matter (WM) changes in Huntington’s Disease (HD) have been shown in a number of studies (see [1], [2] for reviews) and have mainly been studied using diffusion weighted imaging (DWI) and diffusion tensor imaging (DTI). DWI is based on the diffusion of water which is influenced by local tissue properties, and DWI-sequences combine several gradient directions, each of which codes the diffusion along its direction [3] so that DTI characterises the combination of diffusion directions in each voxel. The tensor takes the form of a sphere when diffusion is equal in all directions and a cigar-like shape when a single diffusion direction dominates. The shape information can be converted into compound measures such as fractional anisotropy (FA), a dimensionless scalar ranging from zero (in water) to one, in order to compare groups and to correlate clinical markers with imaging data. In the field of HD research, recent studies indicate that DTI can be useful to measure longitudinal change [4], or to detect early changes in the sensorimotor cortex in premanifest HD [5] so that DTI is an important tool to understand the phenotypical variability seen in HD [6].  

Given these high expectations, it is important to optimise DWI sequences and to perform a rigorous hardware quality control. Physical phantoms play a critical part in large studies such as PREDICT-HD [7] and TRACK-HD [8]. However, subject-related factors are equally important for data quality, particularly in a hyperkinetic movement disorder in which the extent of movement-related artifacts increases as the disease progresses. There is no systematic effect of movement on FA: it can either increase or decrease as a result of movement artifacts. Longitudinal studies need to take the effect of those movements on data quality into consideration in order to remain sensitive to longitudinal change - this is essential to provide biomarkers useful for clinical trials [2].  In the present study, we developed a framework to detect and remove motion artifacts in DWI as an instrument of quality control (QC). Here, DWI data with a high number of different diffusion directions present various challenges including motion-related signal dropouts (cf. Figure 1).  Using DWI data from different stages of HD, we explored a weighted average approach to detect artifacts in two dimensions (slicewise approach): For each gradient direction, the weighted variance was computed from all remaining directions in the sequence by weighting with the angle in which they differ from the index gradient direction. This novel approach was applied to data acquired with different field strengths (i.e., 1.5 and 3 Tesla).

## **Material and Methods**

Data from two scanners were used. Table 1 provides demographic details and the motor scale of the Unified Huntington’s Disease Rating Scale (UHDRS) for the subjects. It also provides the estimated years to the onset of typical motor signs, based on CAG repeat length and age at a 60% certainty level [9]. Given the purpose of this study, we ignored the outcome of a visual data inspection step which would have led to the exclusion of subjects with extensive artifacts. The study was approved by the local ethics committees, and written informed consent was obtained from each subject.

### 1.5 Tesla Data

 Twenty-nine premanifest HD patients and 30 controls were scanned on the same Siemens Sonata 1.5 Tesla scanner (a subgroup of this cohort has been reported in our earlier work  [6]). DWI was performed with an echo planar sequence with a double spin-echo module to reduce the effect of eddy currents [10]. Each data volume consisted of 40 axial slices of 2.3 mm thickness, with no inter-slice gaps, and an acquisition matrix of 96 x 96 in a FOV of 220 x 220 mm^2^, resulting in 2.3 mm^3^ isotropic voxels (inter-slice temporal separation = 155 ms, TE=90 ms, flip angle 90°, fat saturation, bandwidth 2003 Hz/pixel). Each DWI data set consisted of 61 high diffusion-weighted images (b = 1000 s/mm^2^), with diffusion gradients applied along 61 diffusion directions and 7 additional images with minimal diffusion-weighting (b = 100 s/mm^2^). We fit the diffusion tensor using the standard linear least squares fit to the log measurements [11] which also provides an effective b = 0 image. Data acquisition was cardiac-gated to reduce motion artifacts caused by pulsation of the cerebrospinal fluid [12]. Diffusion data acquisition time was 22 min on average, depending on heart rate. An additional T1 weighted MDEFT sequence was acquired (176 slices, 1 mm thickness, sagittal, phase encoding in anterior/posterior, FOV 224 x 256 mm^2^, matrix 224 x 256, TR=20.66 ms, TE=8.42 ms, TI=640 ms, flip angle 25°, fat saturation, bandwidth 178 Hz/pixel) [13] .  

***Table 1: **demographic details for the subjects.*

**Table:** 

	** 1.5 T **	** **	**3.0 T **		
	** Controls**	** PM**	** Controls**	** PM**	** HD**
N (f/m)	30 (15/15)	29 (16/13)	22 (11/11)	23 (11/12)	18 (10/8)
Mean Age (SD)	37.2 (10.0)	40.5 (8.7)	41.7 (7.8)	41.6 (7.7)*	48.8 (8.8)*
Median CAG (Range)	NA	42 (39-47) **	NA	43 (40-47)	NA
Mean years to onset/ disease duration (SD)	NA	16.1 (8.4) **	NA	12 (4.0)	NA
Median UHDRS motor (range)	NA	4 (0-17)	NA	4.5 (0-10)	33 (10-48)
Mean duration of disease (months, SD)	NA	NA	NA	NA	42 (30.0)

 *Significant age difference **exact CAG length missing from two subjects 

### 3 Tesla data

The second group included 22 premanifest, 18 early affected subjects, and 23 controls. All data were acquired on the same Siemens TRIO 3 Tesla scanner. The sequence consisted of 72 diffusion-weighted scans, each with dimensions of 96 pixels x 96 pixels x 55 slices per volume with a 2.3 mm isotropic voxel size; 64 unique diffusion gradient directions (b = 1000 s/mm^2^) and eight b = 100 s/mm2 images; TE was 90 ms. A T1-weighted image was acquired using a 3D MPRAGE acquisition sequence with the following imaging parameters: TR = 2200 ms, TE=2.2 ms (S)/3.5ms (P), FA=10°(S)/8°(P), FOV=280 x 280 mm^2^, matrix = 256x256 with 208 sagittal slices to cover the entire brain with a slice thickness of 1.0 mm with no gap. The 3T MRI scans were acquired as part of the London site TRACK-HD cohort (see also acknowledgment).

### 2-D artifact correction (QC)

We aimed to detect volumes (i.e. gradient directions) with at least one slice showing decreased intensity, i.e. motion artifacts caused by spontaneous subject movement (Figure 1). For each diffusion weighted volume, we first computed the mean intensity for each slice and compared this intensity to the same slice in all other volumes by using a weighted average approach. The contribution to this weighted average was the greater the more similar a given direction was to the index direction. Similarity was defined by computing the dot product between two gradient directions (i.e., a value near 1 reflects great similarity) which we used as a weighting factor. We employed the following scaling procedure separately for each slice \begin{equation*} \Delta I_{ji} = \frac{\left| \bar{a_i} - \bar{a_j}
                        \right| } {\bar{a_i} + \bar{a_j}}\end{equation*} (1)  Here, \begin{equation*}\bar{a_j} \end{equation*} denotes the arithmetic average intensity of the slice under observation and \begin{equation*}\bar{a_i} \end{equation*} a slice for comparison. The relative average intensity deviation \begin{equation*} \Delta
                        I_{ji}\end{equation*} was weighted by the dot product of the gradient direction \begin{equation*}\vec{g_i} \cdot
                        \vec{g_j}\end{equation*}.

If, for one slice of the volume under observation, the average intensity deviation to all other slices exceeded a certain threshold, the whole volume (i.e., gradient direction) from this subject was eliminated. \begin{equation*}\Delta I_j = \frac{1}{N} \sum_{i=0}^{N} \vec{g_i}
                        \cdot \vec{g_j}\Delta I_{ji}\end{equation*} (2) Slices which were not corrupted by subject movement showed values for \begin{equation*}\Delta I_j \end{equation*} (average intensity deviation for one slice of a volume compared to other slices at the same position in other volumes) below 0.2. This threshold was found from a visual inspection of the data and ensured that obvious artifacts were removed. This approach was applied separately to the (b = 1000 s/mm^2^) and (b = 100 s/mm^2^) images in subject-specific native space for each subject. Figure 2 shows an example of this concept for the same subject as depicted in Figure 1.

### Spatial normalization and computation of FA-maps

Subject specific T1 images were first co-registered to the first b=100 s/mm^2 ^image given that its contrast is higher compared to those with higher diffusion weighting. A study-specific template was generated separately for the T1 weighted images at both field strengths (i.e. 1.5T and 3.0T, respectively) using a high dimensional non-linear approach (Diffeomorphic Anatomical Registration Through Exponentiated Lie Algebra, DARTEL) as implemented in the SPM8 software package [14]. We then applied normalization parameters to all DWI from the corresponding subject.  Individual FA-maps were computed from each subject with and without volumes with artifacts removed [11]. A Gaussian smoothing kernel of 8x8x8 mm was applied before entering subjects into the statistical analyses. 

### Whole brain-based statistical analyses

 As we were primarily interested in the effect of image artifacts on the detection of HD specific WM changes, the interaction between group (premanifest HD, early HD, and healthy controls) and QC (FA-maps with and without artifacts removed) was the analysis of interest. Given that substantially less than 1% of volumes had to be excluded from the control groups both at 1.5T and at 3T (Figure 3), the difference between controls with and without QC was negligible. The interaction is therefore reduced to a paired t-test separately for each group of HD subjects with and without QC. To be most sensitive, this analysis was repeated after excluding all cases for which QC did not detect any problems. A lenient threshold of p<0.05 (uncorrected for multiple comparisons) was chosen to ensure a high sensitivity to differences caused by movement artifact. For comparison with previous studies, we also calculated differences between each group of patients and the respective group of healthy controls at a significance level of p<0.001 (uncorrected). 

## **Results**

Figures 1 and 2 show typical scans with movement artifacts and how they are detected in a single subject. Figure 3 displays the frequencies of removed volumes separately for each field strength, diagnostic group, and direction. In the 3T data, more slices were removed from the group of subjects already affected from HD than from either the premanifest or control group. In the 1.5T data, there were more exclusions from the premanifest group than from the controls.

### Effect of QC

 Paired-t-tests of premanifest and early patients with and without QC did not show significant differences even at a lenient threshold of uncorrected p<0.05. The effect of QC for FA maps of single subjects (Figure 1, bottom section) is usually < 0.1 (see also Discussion). 

### Group comparison

The findings of the between-groups comparison mirror those reported in previous studies [15] [16]. Premanifest subjects show increased FA values in the striatum while FA values in control subjects are higher at white matter/CSF boundaries (Figure 4). We also observed increased FA values in the corpus callosum of control subjects when compared separately to premanifest and early HD subjects scanned at 3T. 

**Figure:**
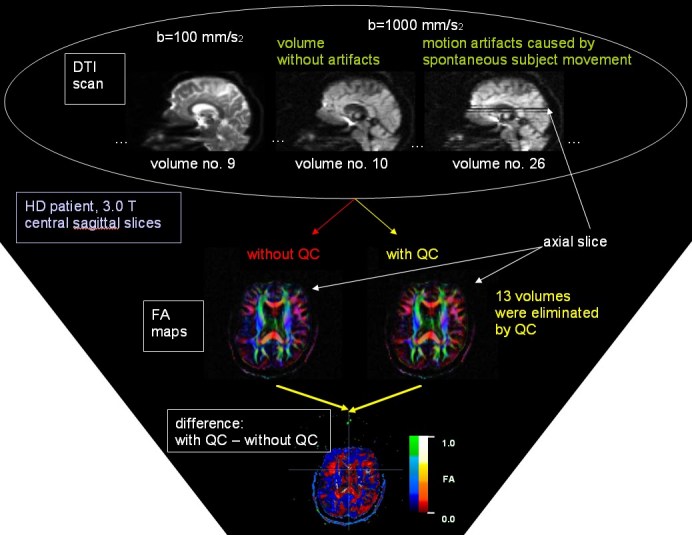
***Figure 1:** Schematic flowchart of quality check (QC) data processing for an example subject (HD patient at 3T). **Upper panel: **series of sagittal slices with and without motion artifacts. The QC algorithm eliminates the respective volumes (i.e., vol. no. 26 and twelve further volumes for this data set). Differences in FA maps calculated without and with QC (**central panel**) could be visualized (**lower panel**). *

**Figure:**
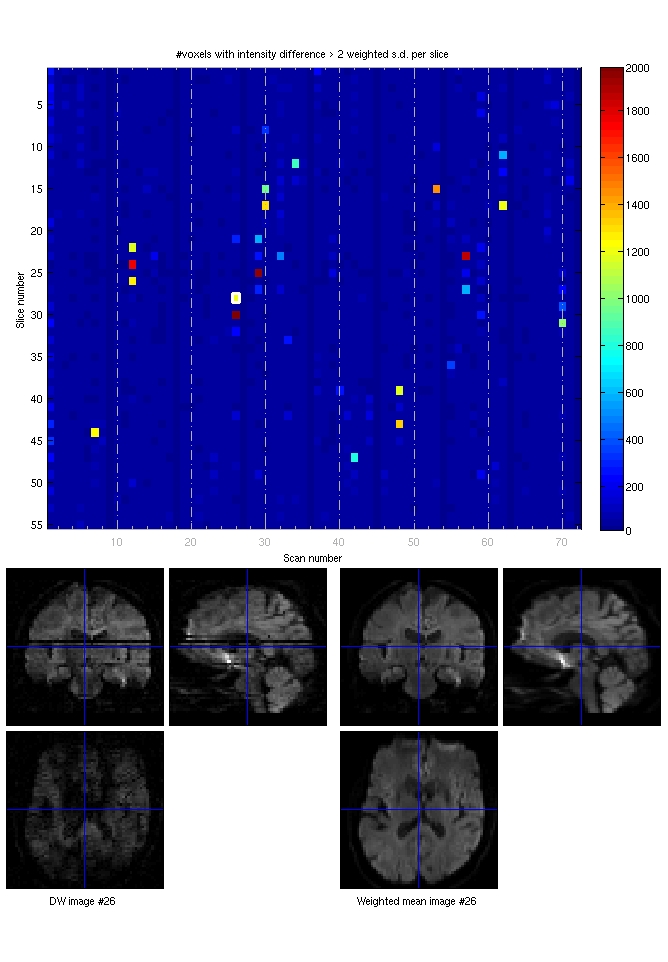
***Figure 2**: DTI QC review tool. Upper panel: For the same subject as in Figure 1, the difference to the weighted average is shown for every volume (x-axis) and every slice (y-axis). The color codes the number of voxels with more than 2 standard deviations from the average.** Lower panel**: A DWI with substantial drop out artifacts is shown on the left. It can be detected automatically by comparing it to the weighted average (**low right panel**). *

**Figure:**
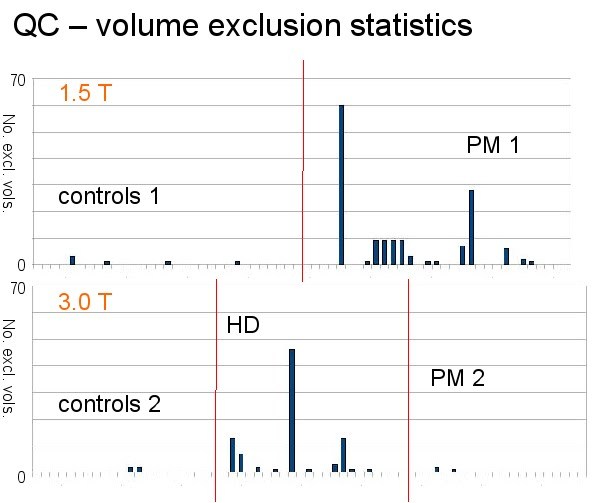
***Figure 3:** The number of volumes excluded in the QC process are displayed separately for each subject and for each of the diagnostic groups: HD – early affected Huntington disease patients, PM – premanifest HD patients, and controls; the numbers 1 and 2 refer to the field strength (1 = 1.5T; 2 = 3.0T). *
 

## **Discussion**

In order to study the effect of DWI artifacts on HD specific WM changes, we developed a QC framework to detect and remove motion artifacts in DWI. Many tools have previously been developed for QC in functional MRI (e.g. TSDiffAna; http://cibsr.stanford.edu/tools/ArtRepair/ArtRepair.htm or ArtRepair Toolbox; http://sourceforge.net/apps/trac/spmtools/). Also big efforts have been performed in the National Alliance for Medical Imaging for data cleaning (http://www.nitrc.org/projects/dtiprep/). These tools focus on spike artifacts caused by technical equipment in the scanner room or by the scanner itself, based on the assumption that each image in a series should look similar to the other images. To some extent, these approaches can be used on DTI analysis as well but those presented here (or e.g. by the National Alliance for Medical Imaging) seem more appropriate as DTI data present multiple additional challenges. Image contrast depends on strength and direction of diffusion weighting so that, in any two images, voxels in the same anatomical location may have completely different intensities because of local diffusion properties. Therefore, two images from the same DTI series can be compared directly only if they are acquired using the same diffusion weighting and direction. In addition, there are motion related signal dropouts (Figure 1) which usually do not occur in sequences without diffusion weighting. Given limited scanning time, most current DWI sequences are designed to include a high number of different diffusion directions rather than fewer directions multiple times. This has the advantage that the local diffusion tensor can be more accurately described but limits artifact detection software based on classic similarity measures. 

**Figure:**
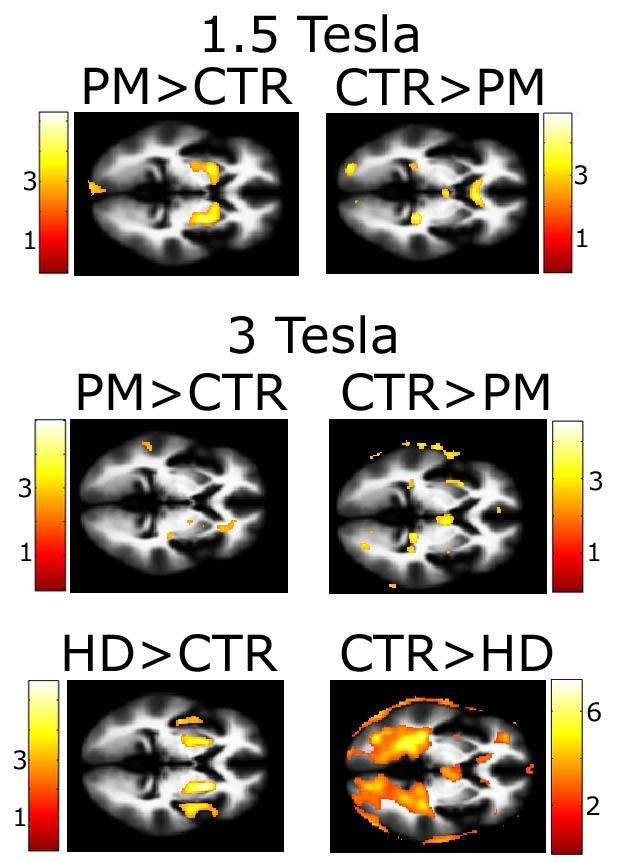
***Figure 4: **For comparison with previous studies, FA maps of pre-symptomatic and early HD cases were compared with healthy controls. Results are overlaid on the scanner specific template. Color bars display T-scores for each of the comparisons. HD, early affected manifest Huntington disease patients; PM, premanifest mutation carriers; CTR, controls.*

 The effect of QC on single data sets can be estimated to be in the range of the ratio of eliminated volumes to the total number of volumes; here in this study due to a high number of gradient directions for single subjects and a moderate number of eliminated volumes (Figure 3) this effect is low (usually FA differences < 0.1). Nevertheless, in order to give a rough estimation, as over 60 (b = 1000 mm/s^2^) directions and also more than 5 (b = 100 mm/s^2^) data sets were recorded, the exclusion of less than e.g. 10 data sets (which is the case for most data sets, Figure 3) generally could lead to changes in single FA-maps of about 10-20 %, as the process of Eigenvector/Eigenvalue calculation and subsequent FA calculation follows the rules of a linear process. Therefore, the changes in FA values (without and with QC) cannot be expected too high for single data sets. Subsequent group comparison (group strength about 20) equalizes the remaining outliers.  Therefore a high number of gradient recordings helps to improve the quality of the results. If the ratio between eliminated volumes to the total number of volumes is high, QC could act as a tool to improve the quality of single subject results and consequently also improve the results at the group level. Thus, beside signal accumulation, increasing the number of gradients and further possibilities, QC is an additional tool to increase the signal-to-noise ratio in DTI data analysis in order to improve the quality of the results in group comparison. We present a novel approach for the QC of DWI data by use of both 1.5 and 3T data as the currently used standard field strengths in MRI studies of HD. This QC detection method is suitable for the current DWI sequences that employ a high number of unique directions rather than multiple times scanning fewer directions. The results of the data analyses support the current literature of DTI applications to HD [16], i.e. both significance levels and the regional distribution of HD-associated FA alterations were in accordance with those previous studies.  As an additional indicator of plausibility of the results, more volumes were excluded as the disease progressed, i.e. more data were excluded for HD in comparison with controls. This is not unexpected for neurodegenerative movement disorders, but other factors such as increased anxiety may also have contributed to this effect. The threshold effectively controls the trade-off between an unnecessary loss of data by being too conservative and including too much noise. The cut-off chosen in the current study was selected through visual inspection of the images. Although this seems to be somewhat arbitrary, it has to be held that when varying this threshold between 0.2 and 0.3 as detailed in the methods section, almost the identical slices were detected.  Several extensions and alternatives to the current implementation are possible, starting at the level of the preprocessing. We refrained from performing a rigid body registration of the DWI data. Registration is difficult for images with high b value and differing gradient directions. The interspersed images with low b value could be used but require strong assumptions regarding the type of movement that occurred. Moreover, this study was restricted to FA measures, the extension to more complex DTI parameters such as fiber tracking, which are more sensitive on an accurate diffusion tensor and thus the number of gradient directions, has to be topic of future studies.  Although it should be kept in mind that excessive movement and other artifacts increase noise and will therefore decrease the sensitivity to detect true artifacts, the results presented here are encouraging for large-scale studies in HD. Despite obvious movement in several cases, these movements did not influence the FA value systematically. These results do not differ when the QC tool is included into the postprocessing, even when the statistics are leniently uncorrected. This speaks to the relative robustness of the FA values in DTI research in HD which are computed by taking all directions into account. Given a thorough postprocessing of the MRI data as previously demonstrated in morphometric T1 weighted MRI analysis [17], this observation, as a further conclusion, speaks to the potential of the use of MRI-based measures such as DTI as a biomarker in HD  

## **Acknowledgement and Funding**

This work was supported by the European HD network (EHDN project 070). MN is funded from a Wellcome Trust grant held by ST (075696/Z/04/Z). The 3T MRI scans were acquired as part of the London site TRACK-HD cohort. TRACK-HD is supported by the CHDI Foundation, a not for profit organization dedicated to finding treatments for HD. The authors wish to extend their gratitude to the London TRACK-HD study participants and to Beth Borowsky, scientific director for TRACK-HD at CHDI. Some of this work was undertaken at UCLH/UCL who acknowledge support from the respective Department of Health's NIHR Biomedical Research Centres.

## **Competing interests**

The authors have declared that no competing interests exist. 
